# Taxonomies of exclusion: Storytelling, naming and classification in an age of extinction

**DOI:** 10.1017/ext.2024.7

**Published:** 2024-04-01

**Authors:** Eline D. Tabak

**Affiliations:** Faculty of Humanities, University of Oulu, Oulu, Finland

**Keywords:** cultural extinction, nomenclature, classification, poetry, ecocriticism

## Abstract

Among the stories on individual examples of charismatic fauna, there are also extinction stories that evoke databases and their aesthetics in how they list endangered species. At the same time, these different stories grapple with a legacy of taxonomy that, while necessary in conservation, also carries a history of exclusion. This paper turns to the poetry of Claire Wahmanholm and Juliana Spahr to consider some of the ways extinction stories can be told outside of the relatively narrow scope of charismatic species. To begin, I reflect on extinction storytelling and the classificatory impulse in some of these stories, including poetry. Then, I consider scientific practices of naming before I turn back to Wahmanholm and Spahr and explore practices of naming and classification in their poetry. Following that, I dwell on the influence of scientific classification on the ways people including poets can engage with extinction. The poems in this paper are not merely an object for analysis; they should be considered an invitation to come to terms with and move beyond complicated histories and practices of naming and classification in storytelling.

## Impact statement

This article looks at how practices of scientific classification influence and limit engagement with endangered species in research and culture, specifically poetry inspired by extinction lists. It is important to understand the role and extended influence of taxonomy and classification when it comes to extinction and the ways people engage with endangered species by employing an interdisciplinary lens. Extinction is a biocultural issue; the problems connected to taxonomic bias can be seen in far more than conservation efforts. Cultural expression gives deeper insight into the influence of scientific practice and its limitations on conservation efforts, which is necessary to address all elements of the current extinction crisis.

## Introduction

Extinction storytelling largely focuses on megafauna, often mammals and birds, that have become culturally important (Heise, 2016; Jørgensen, 2019; Pyne, 2023). While there are exceptions, like eels, snails or even unknown extinctions, this does raise the question of why it is that, in an age of overall biodiversity loss, the public imagination is still predominantly fixated on a few select species.^1^ Recent research shows that cultural and societal aspects factor into species extinction as well (Ladle et al., 2023). In their work on extinction stories and histories, Heise (2016) and Jørgensen (2019) argue that endangered and extinct animals come to matter once their lives become part of a human history and the stories people tell. On top of that, media coverage and creative writing on extinction often are limited to birds, mammals and other large animals, known as charismatic, flagship or emblematic species (Bowen-Jones and Entwistle, 2002; Lorimer, 2007; Berti et al., 2020). Examples of these include endlings such as Martha the passenger pigeon and the last thylacine, as well as charismatic animals like polar bears, whales and a variety of big cats (Jørgensen, 2017; Albert et al., 2018). Thus, despite growing awareness that other species might require attention before it is too late, the cultural imagination is still pointed to a well-known and often storied group of animals in an age of overall biodiversity and biomass loss.

But with thousands of species at risk, the ways in which extinction is approached and storied are changing. Taxonomic bias in conservation, research and the stories shared, whether in media, literary narratives or even schoolbooks (Clark and May, 2002; Heise, 2016; Gangwani and Landin, 2018; Forster et al., 2023), exemplify how these issues are entangled and in need of a transdisciplinary perspective to tackle them. Indeed, authors are responding to the impetus to look beyond charismatic and flagship species and share stories across different forms of art that respond to the sixth extinction crisis. Inspired by these new stories, this paper takes poetry as its point of departure to look at how extinction storytelling can both look beyond the narrow narrative of charismatic species and still grapple with the legacy of taxonomy that, while most definitely necessary, also carries a history of exclusion. Extinction storytelling has a taxonomic bias problem, but the problem goes deeper than a preoccupation with certain species.

To begin with, this paper will briefly reflect on extinction storytelling and poetry before it moves on to this “deeper” problem: the new natural history. Second, I will consider the onto-epistemological and ethical implications of scientific practices of naming. With naming, especially, the ontological relates to the epistemological. That is, ways of knowing the world are entangled with being or existing in the said world, which in turn makes onto-epistemology an ethical matter (Barad, 2007). Then, I will turn to Claire Wahmanholm and Juliana Spahr’s poetry and explore how their poetry uses naming and listing. Following that, I will move on to the influence of scientific classification as exemplified by the analysed poems. My analysis focuses on overlooked uncharismatic animals and new ways of engaging with endangered and extinct species, and so lingers on the class *Insecta.* The poems in this paper serve not only as an object for analysis but also as an invitation to contemplate these histories, specifically considering practices of naming and classification, and how to move beyond them. Taxonomic bias in both research and broader cultural expression, such as extinction storytelling is very much a sociopolitical and cultural issue and cannot be changed by looking at the fields of biology and conservation alone.

## Extinction storytelling

Storytelling, as van Dooren and Rose (2016) write, ‘is one of the great arts of witness, and in these difficult times telling lively stories is a deeply committed project, one of engaging with the multitudes of others in their noisy, fleshy living and dying’ (91). Yet, with certain narrative practices following and foregrounding the supposed order of natural life rather than unruly, situated or indeed “lively” stories, it can be challenging to forge such a deep commitment to this project. Furthermore, certain species are disproportionally represented among creative narratives of decline because they are part of longstanding cultural histories and speak to the imagination. Telling the story – or rather, *one of the stories –* of extinction is not an easy task, especially when it comes to animals most people only encounter in zoos or museums, or, indeed, less charismatic groups of animals like insects. Although people have certainly made attempts to do so (Rose and van Dooren, 2011). Extinction storytelling comes in many forms: literary, visual, auditory, long and short. Out of all these different forms and genres, I turn to poetry not just for the relative flexibility of its form and content (although this greatly differs with poetic genres, traditions and individual poets), but mostly as poetry can be a place where writers ‘defy, distort and transform their everyday language into … acts-against-extinction’ (McCabe, 2019, 3). The poetic not only reflects but also creates.

When it comes to extinction storytelling, I am especially interested in what different forms, including poetry, do in today’s era of information overload. More specifically, what poetry can do among what Houser (2020) calls “infowhelm”, a term that refers to the ‘abundance and ready availability of information as well as its contestation’ (1). Instead of redirecting attention to less charismatic species like eels or snails, the significant amount of available information also allows for different ways of engaging with species loss. Extinction is inventoried, tracked and listed across several media. Vast quantities of information on extinction are available and shared through a variety of media, including art that follows the new natural history’s classificatory impulse. This impulse is most certainly present in the poems below, where species gain recognition in the list only because of their endangered or extinct status. But, as I hope to show throughout this paper, onto-epistemologies are everywhere, including in the seemingly objective shared information *and* the ways it is shared. That is, knowledge production affects our understanding of all beings on Earth and specific beings.

It seems only natural that people turn to other means of telling the story of wildlife or insect decline when not focussing on specific and highly individualised charismatic species. Examining the poetry below, I argue that instead of fixating on and naming individuals, storytellers turn to multitudes: in particular by naming species and making lists. As I show, this turn to multitudes through a very specific poetics provides a contrast to popular extinction storytelling about named individuals. Examples include endlings like Lonesome George and Martha the passenger pigeon (Jørgensen, 2017). As named and well-known endlings of their species, these singular animals have a noticeable presence in extinction histories and storytelling. They all come with their own sociopolitical histories, and with that, *stories.* As such, they carry with them the burden of (cultural) grief. The move from naming individuals to naming species, like the poems discussed in this article, removes this burden from specific individuals and instead draws attention to extinction at the species level. I explore how the abecedarian poetry of Wahmanholm and Spahr approaches this artistic move to the polyphonic below.

‘Lately my dreams have been more dead / than usual. They have always been a little dead / but only around the edges’ (ll. 1–3). These first lines of Wahmanholm’s poem ‘Deathbed Dream with Extinction List’, published in the collection *Meltwater* (2023b), subtly emphasise that extinction is becoming an increasingly integral part of people’s daily experience. Throughout the rest of the poem, Wahmanholm lists (presumed) extinct flora and fauna, beginning with a plant: the ‘*Appalachian yellow asphodel*’ (l. 6). The poem is an abecedarian. Traditionally, the abecedarian form marks a poem in which the first letter of a line or verse follows the alphabet. In ‘Deathbed Dream’, the names are dispersed freely throughout the poem and sentences rather than at the beginning of a line or verse. Wahmanholm uses the letters of the modern English alphabet to create a structure in the poem that takes the form of a list. It ends with the ‘shaky song of the Zulu ambush katydid’ (l. 68), a bush cricket. Except for a few examples, like the Hawai’ian Kauaʻi ʻōʻō, all animals are referred to by their English common name. The use of an abecedarian list in Wahmanholm’s poem is reminiscent of Spahr’s ‘Unnamed Dragonfly Species’ (2011), a 19-page long poem that lists just under 150 different species of fauna in bold font that starts with the letter a, skips the x and ends with a single letter y: the yellow-breasted chat. Ending the poem with just one y-lettered animal can also be interpreted as a sign that the list of extinct and endangered species is far from complete. It could also simply be a matter of practicality – there are not that many animals with English common names that start with an x, y or z. These extinction poems reveal an inclination to name species and list them. When it comes to cultural explorations of extinction, it appears that the names are important. These poems are part of what Houser (2020) calls the *new natural history*, ‘a prevalent but overlooked trend within the contemporary arts in which natural history provides both theme and method for cultural production’ (17).

As examples of poems inspired by the new natural history, Wahmanholm and Spahr’s poetry will be the main focus of this paper. The poems ‘Deathbed Dream with Extinction List’ and ‘Unnamed Dragonfly Species’ exemplify that some ways of storytelling are built upon a system that has become universalised, even considered natural, even though it is as determined by sociopolitical circumstances as any other. The universal acceptance of this system as a way of ordering natural life is also reflected in narratives of species decline – and wider storytelling practices on endangered, threatened and extinct species, including databases like The IUCN Red List of Threatened Species (2021). As inspired as these narratives are by the supposed order of natural life, they also uphold the onto-epistemological hierarchies present in these orders and restrict the ways in which people are invited to engage with species decline and biodiversity loss. In art (both visual and literary), this trend ‘follows the classificatory impulse and its epistemological and representational traditions to unleash similarly productive failures’ (103). These productive failures include a history of taxonomic bias.

## The problem with names

To begin, I want to dwell on the ethics and onto-epistemological implications of naming. Practices of naming come with their own, to use Barad’s (2007) terminology, ethico-onto-epistemology. Meaning that when it comes to knowledge production and dissemination, ways of being in and knowing the world are inseparable from ethics. The naming of animals (and other organisms, such as plants and fungi) has a longstanding but not uncontested tradition in Western literary and scientific history (Borkfelt, 2011). One of the oldest examples is found in the Old Testament, specifically Genesis 2:20, in which Adam names all the animals. Current naming practices are a little more complex, although they can also be traced to a single man. Many species have common and scientific names. In the eighteenth century, Swedish taxonomist Carl Linnaeus formalised and popularised binomial nomenclature, the formal two-term naming system by which all living organisms are named. These binomial or scientific names are always written in Latinised form, although the words can be derived from other languages, and consist of a generic name, identifying the genus, and a specific name or epithet, distinguishing the species. Linnaeus’ nomenclature replaced an older polynomial system heavily focussed on description, and the effectiveness and singularity of the binominal system ensured its popularity and widespread use.^2^ This does not, however, mean that the binominal system was accepted everywhere from the start.

In *Plants and Empire*, Schiebinger (2004) quotes Linnaeus’ disgust at the ‘chaos and confusion’ and ‘barbarity’ of modern naming of organisms (194–95). Linnaeus’ own preference for Greek and Latin and apparent disregard of other naming traditions and systems can, as Schiebinger also writes, easily be identified as a form of linguistic imperialism, ‘a politics of naming that accompanied and promoted European global expansion and colonisation’ (195). In this case, colonisation transpired through the universalisation of a Eurocentric naming system for the rest of the world, which in turn amounted to a specific form of scientific imperialism. Notwithstanding the colonial and at times violent history of science, naming organisms and describing the world is important, if only to give a face to other-than-human animals and to promote a better understanding of anthropogenic environmental change and the breadth of the sixth extinction. Simply put, naming practices are political.

For example, Linnaeus’ binominal system went beyond a preference for the classics. As Lafuente and Valverde (2007) write, Linnaean botanical nomenclature in the Spanish colonies ‘also served as a political “nomenklatura” insofar as the exclusion of native names from the field of science defined new power relations. Linnaeus’s nomenclature acknowledged the authority of imperial botanists and belittled local herbalists and herbal practitioners’ (137). Lafuente and Valverde speak of scholars in both the old and new worlds who candidly wrote about the faults with Linnaeus’ system, which they found ‘characterised by its insensitivity to local circumstances’ (*ibid.*). Schiebinger also notes Linnaeus’ exclusionary botanical naming practices, in particular the choice to ‘celebrate botanists known to him – a practice that reinforced the notion that science is created by great individuals, and in this case European men’ (3). By doing this, Linnaeus’ popular system successfully rewrote scientific history. The history of Linnaeus’ naming practices, or the ‘story of elite European botany’ (*ibid.*), that Schiebinger so carefully recounts here is a crucial example of linguistic imperialism. This highlights that the exclusionary politics of the taxonomic system are not just present in classifications created in the said system, but also in the very names of scientists in positions of authority who decided to give new and old species. Rewriting local histories through naming – and with that heterogeneous knowledge and classification systems – is not exclusive to the history of taxonomy (Plumwood, 2003). The European colonial project caused histories around the world to be rewritten in ways that privilege Western scientific expertise over other communities, including practices of naming (Mabele et al., 2023). Renaming places and species is not innocent and can be considered an epistemological act of violence. These implications invite a closer examination of extinction stories with a foregrounded practice of naming.

## Extinction poetry

In the face of extinction, taxonomic practices have also become part of new forms of poetry. Perhaps the most famous of these is Juliana Spahr’s work, the poem ‘Unnamed Dragonfly Species’ in the collection *Well Then There Now* (2011). Spahr’s prose poem has been widely discussed as an exemplary poem that ‘brings the quantitative and experiential aspects of climate change to the page through an imaginative poetics that captures mundane data encounters in our current climate emergency’ (Houser, 2020, 22–23). The poem ‘Gentle Now, Do not Add to Heartache’ from the same collection also lists species and other natural – and unnatural – phenomena. While Spahr herself has called it an anti-capitalist poem, she also concedes that it is often read as an elegy (Goldsmith, 2016). With a much more foregrounded elegiac message, ‘Unnamed Dragonfly Species’ takes stock of biodiversity and commemorates the listed species. Interspersed through a long narrative poem that touches upon pollution, melting glaciers and current and future environmental refugees, are animal names. This list is an intervention: the names are alphabetised, printed in boldface letters, capitalised and there is no punctuation between the animals’ (common) names and the following sentence. It forces the reader to stop and take stock:

The city of Rotterdam sent over daffodils. **A Noctuid Moth** The daffodils bloomed in the first weeks of April. **Allegheny Woodrat** They were everywhere. **American Bittern** They were yellow.

(75)

The effect is immediate: what do noctuid moths have to do with daffodils and Rotterdam, let alone with the Allegheny Woodrat and the American bittern? Interestingly, the first animal on the list only names a particular insect family. The author decided to put the article “a” in front of what is commonly known as the family of owlet moths, and apart from ‘**A Noctuid Moth**’ (75) the ‘**Unnamed Dragonfly Species**’ (92) in the final stanza, all are specified as common animal names. One cannot help but notice that both examples – an unspecified member of the owlet moth family and an unnamed dragonfly species – belong to the class *Insecta.* ^3^ In a list of 149 different animals, these two unspecified insects are conspicuous and show taxonomic bias even among practices of naming, especially in taxonomic classification, which is so important in understanding extinction and aiding conservation efforts.^4^ Thus, the preoccupation with naming in creative narratives, as I argue throughout this paper, draws attention to certain aspects of naming practices in scientific practice as well.

Additionally, every other page (the right on the printed version) of ‘Untamed Dragonfly Species’ ends with an animal name and no punctuation, and the reader must turn the page before the poem continues. As mentioned above, the poem ends with the letter y, or the yellow-breasted chat, instead of z. There are enough (sub)species of animals that Spahr could have ended the poem with the final letter of the alphabet. Instead, the poet leaves the reader with an unfinished list. Similar to the intervention of the different animal names throughout the poem, the reader is asked to pause. What is the intention of ending the list before the final letter of the alphabet? Perhaps here, Spahr means to draw attention to the impossibility of cataloguing life, or to the never-ending list of species affected by the numerous environmental crises discussed in the narrative part of the poem. At the same time, by not completing a catalogue of endangered species, the author could very well try to convey a small message of hope.

Claire Wahmanholm’s poetry collection *Meltwater* (2023b) also gives in to the classificatory impulse of the list. There are two poems dedicated to two letters: ‘M’ and ‘P’. The poems sit somewhere between an abecedarian and a tautogram, and both list animals, verbs and other phenomena starting with their respective letters. ‘M is for the migrations of monarchs, mule deer, / mullet, for magnetic fields, for the way the world pulls you from me and you / materialise’ (17, ll. 11–13). It seems that this particular enumeration combines verbs with animal species with attraction for no other reason than all these words start with the same letter: an alphabetical classification. Here, I cannot help but be reminded of Foucault’s reading of Borges’ work; the implied categories of these lists, as arbitrary as they seem at first glance, can all be assigned ‘precise meaning and a demonstrable content’ (Foucault, 2002, xvi). Another of the collection’s poems, ‘Glossary of What I’ll Miss’ (62–63), is a true abecedarian: the first letter of each of the 26 lines sequentially follows the English alphabet. The lines themselves are divided into neat stanzas of two: ‘Autumn, always. The buzz / by which we know the katydid and fly’ (62, ll. 1–2). The extinction abecedarian is reminiscent of the distinctive grammar of climate change, which prepares for what is not yet gone but soon might be (Garrard, 2016, 297). All species listed in Wahmanholm’s ‘Glossary’ are presented as beings that will come to pass, placing this poem in an anticipatory category for all that will be lost in the future.

Also responding to the new classificatory impulse, the poem ‘Deathbed Dream with Extinction List’ interweaves 26 species in alphabetical order within the poetic form. The poem is not an abecedarian in the traditional sense, but certainly follows the concept. Wahmanholm has said the following of this poem, which imagines a deathbed dream in which the speaker is visited by extinct species: ‘I realised that I could not think of one extinct species per letter of the alphabet. I had to look up and get the character of each of the species that appears in the poem’ (Wahmanholm, 2023a). The listing of species is intentional and required research in order to complete the full alphabet, which is an act of careful attentivity to Wahmanholm: ‘an act of care, an act of recovery’ (*ibid.*) Only the first animal, an Appalachian yellow asphodel, is italicised: ‘*Appalachian yellow asphodel*, the dream says,’ (2023b, 78 l. 6). But this act of care also draws attention to the particular poetics of extinction databases and lists, which comes with a value judgement. That which is most vulnerable, or even already extinct, is considered to be the most valuable. Wahmanholm plays with this idea of value as well:

Long enough to erase the large sloth lemur and the Mariana mallard and eighty-three per cent of everything else. On balance, it’s unnatural to be living. A statistical impossibility. *Extinct* means *extinguish* means *quench,* but what mouth is so thirsty for our deadness? (ll. 31–36)

The 83 % cited in the poem refers to a number recently published on how much mammal populations have decreased due to human activity, although it should be noted that the poem also includes plant species. Listing and naming prove to be powerful techniques when it comes to creating poetics of extinction. But with the unspecified noctuid moth and the unnamed dragonfly species highlighted in Spahr’s work, and Wahmanholm having to look for names to fit an abecedarian, one cannot help but wonder if the classificatory impulse found in these poems also successfully includes less charismatic animals like insects.

## The ethics of classification

Poetry that responds to the classificatory impulse, then, upholds a taxonomy of exclusion. In order to fully understand this, a closer look at classification and where it comes from is necessary. The current system of classification influences not just research but also culture, and with that the ways with which people engage with the natural world around them and ultimately approach extinction and conservation. These scientific developments have a history of power, colonialism and onto-epistemological exclusion. At the same time that European scientists asserted dominance of the natural world by following the new scientific method, related forms of knowledge production – whether by women, indigenous peoples and other non-western communities – were considered to be incorrect and unscientific as they did not fit the new epistemological paradigm (Pratt, 1992; Plumwood, 2003; Huggan and Tiffin, 2010).^5^

While the roots of our current environmental crises are often attributed to developments originating in the Enlightenment period, Plumwood (2003) argues that this is a misconception that ‘fails to recognise how deeply rooted in the western tradition is the oppositional account of reason and the associated master account of human identity and denigration of nature’ (75). Still, this period did see, as Plumwood continues, ‘a major intensification of the domination of nature, just as our own period involves a major intensification of the instrumentalisation of biological life’ (75).^6^ I mention the significance of the *intensification of the domination of nature* present in the Enlightenment specifically as it coincides with major developments in biology and taxonomy, including new trends in the naming and classification of species. As Foucault (2002) argues, the main objective of the Enlightenment was to put all of life into logical order, or to ‘tame the wild profusion of existing things’ (xvi), which was achieved by introducing and refining new categories. The categorisation so characteristic of modernity (including its amplification in the sciences in the eighteenth century) is present until today.

And yet taxonomic classification helps to create an overview and shape knowledge of what species are out there and how well their populations are doing. Classification and knowledge of species’ conservation status allows for priorities to be identified, create appropriate conservation strategies and look into alternatives when necessary. In short, the importance of taxonomy for conservation is undeniable (Dubois, 2003; Peterson, 2006; Vogel Ely et al., 2017). In the context of extinction, the necessity of categorisation is twofold: taxonomy or the classification of organisms and the different categories or ranking in Red Lists such as the IUCN database.

The theory, practice and subsequent *rules* of classification are the results of the scientific paradigms in which they were developed. This means that rather than objective or static categories, classifications have changed over time and have been adapted according to new discoveries in the field. They are influenced by socio-cultural practices and norms, ethics and politics, and in turn influence those. ‘Classifications’, write Bowker and Star (1999), ‘are powerful technologies […] perceived as real, [they have] real effect’ (319). In a time where, using Plumwood’s words, the mastery of not just nature, but also women and people of colour intensified, it comes as no surprise that the emerging scientific practice reflected the ethics and politics of dominion. Similarly, taxonomy comes with its own ethico-onto-epistemology. Decolonial feminist scholar Lugones (2010) writes that the ‘categorial, dichotomous, hierarchical logic (is) central to modern, colonial, capitalist thinking about race, gender and sexuality’ (742). But this logic does not stop at race or gender. Where Plumwood highlights the connections between the oppression of both nature and women, Lugones argues that the ‘colonial imposition of gender cuts across questions of ecology, economics, government, relations with the spirit world and knowledge, as well as across everyday practices that either habituate us to take care of the world or to destroy it’ (742). These everyday practices have come to include naming and classification as two ways of engaging with extinction. These systems of knowing and seeing the world are so dominant that they limit our engagements with life to the extent that they seem to be the only way to knowing others. And as discussed in my reading of Spahr and Wahmanholm’s abecedarian poetry, even attempts to cover the breadth of extinction leave out certain forms of life when using an exclusionary system.

Following the classificatory impulse of endangered species, the poems ‘Deathbed Dream with Extinction List’ and ‘Unnamed Dragonfly Species’ also exhibit the aesthetic of the database (Vesna, 2007), which is perhaps unsurprising considering the significant cultural impact of the IUCN Red List. Databases turn heterogeneous sources into a homogeneous source of information. For the sake of homogeneity, this requires conflating all species’ information. A provisional look at the IUCN database shows, in a neat square, the species’ photo, their taxonomic information, common and scientific names (the former only if they have one), and their red list category. It is a surprisingly sterile and unattached way of engaging with life at the edge of extinction – just like the alphabetical lists in Wahmanholm and Spahr’s poetry. The species listed are deprived of all context: knowledge of their social lives, habitat and more. On the one hand, databases and extinction poetry like the examples above, grant species individuality by naming them. Naming and listing renders them visible (no matter how shallow) in ways that they were not before, especially when it comes to animals and plants that are difficult to individualise, like insects. In direct contrast with more elegiac and tragic traditions, databases also come with the potential to desentimentalise and deromanticise mass extinction by offering a more panoramic view of biodiversity loss (Heise, 2016). This is especially salient when it comes to insects (like the unnamed moth and dragonfly species in the poems discussed above), whose unmatched multiplicity renders them particularly difficult to connect with (Kellert, 1993). Considering all this, the narrative the database constructs is exclusionary. The very act of organising information effectively homogenises all life and ignores and erases the biocultural worlds of which each species is a part.

## Conclusion

Naming and classification are two inextricable parts of producing and sharing taxonomic knowledge. They are instrumental in understanding the spread and complexity of the biodiversity crisis in scientific contexts and beyond, like extinction storytelling. While a necessity, this does not mean that naming practices and taxonomic classification and the stories inspired by them are without fault. Practices of naming influence both scientific knowledge and cultural expression, or what Houser calls the new natural history, highlighting the importance of a transdisciplinary perspective on practices of naming in an age of overall biodiversity loss. Taking all of this into consideration, this paper is, broadly speaking, concerned with how taxonomy, specifically naming and classification influence storytelling on extinction and biodiversity loss. Using poetry as an example, it does so by looking at practices of naming and taxonomic classification. The poetic highlights issues with taxonomic naming and exemplifies how pervasive these histories are when trying to engage with extinction and biodiversity loss beyond the scientific. As the authors of *Arts of Living on a Damaged Planet* write, people ‘often tally the plants and animals at risk of extinction one by one on lists of endangered species. But’, they continue, ‘single species are not the best units through which to see extinction – because they are not the units of life’ (Tsing et al., 2017, m141). While abecedarian poetry like Spahr and Wahmanholm’s extinction lists gives both authors and readers the opportunity to move beyond charismatic species, a close reading of these poems foregrounds the problematic history of what Mabele et al. (2023) call the ‘epistemic empire’. A new way of looking at, sharing information, and ultimately connecting with other-than-human life is necessary. This is not the task of scientists and academics or poets and storytellers alone. New artistic collaborations and transdisciplinary research could be the solution to a problem pervasive in both research and cultural expression on extinction.

## Footnotes

1 Some of these animals are umbrella species, and their protection indirectly positively and affects other species as well. Rose and van Dooren (2011) edited a special issue on what they termed “the disregarded”, which includes essays on moths, mushrooms, and flying foxes. For examples of other stories (both academic and non-academic) on non-charismatic or unseen animals, see Bastian (2020) on whale falls and unknown extinctions, van Dooren (2022) on snails, and Malay (2023) on eels.

2 These names, too, tell a story. Some stories are long and others are short. For example, specific names are sometimes inspired by the people who supposedly discovered them, or they describe the unique appearance of the organisms, or can gesture towards pop culture and contain in-jokes between colleagues. The use of eponyms in biological nomenclature is also the focus of discussion among scientists (Guedes et al., 2023; Jost et al., 2023).

3 Houser argues that the unnamed human voices of the poem, ‘They’, also belong in the list of unnamed animals.

4 It is currently estimated that only 5 % of insects are described per today’s scientific standards – and thus named. Looking at the IUCN Red List, a well-known example where we also find taxonomic bias, recent research by Manu Saunders et al. on the state of the world’s wildlife has shown that, out of an estimated one million described species, only 8,131 are assessed on the Red List (Saunders et al., 2023). This is only 0.16% of the million described species. On top of that, population trends as seen on the Red List mark 75.5% as unknown, 10% as in decline, 0.5% as increasing, and 14% as stable. This 10 % is significantly lower than the 28% decline in assessed species that the IUCN 2021 proclaims on their homepage. When it comes to insect species and their decline and increase, there is work to be done on ‘the barometer of life’.

5 The process of the control of other-than-human nature, and concurrently women and people of colour, is not unique to or began in what is now considered to be the Enlightenment period, or even modern history.

6 For the sake of brevity, I will confine my analysis to the Enlightenment movement and modernity.
