# Supplementary material for: Taxonomies of exclusion: Storytelling, naming and classification in an age of extinction
Source: Camb Prism Extinct. 2024 Apr 1;2:e4. doi: 10.1017/ext.2024.7 (PMC11895716; doi:10.1017/ext.2024.7)
Supplement: Peer review history

# Editor report: Recommendation: Taxonomies of exclusion: Storytelling, naming and classification in an age of extinction — R0/PR2

**DOI:** 10.1017/ext.2024.7.pr2

**Affiliations:** School of Languages, Cultures and Societies, University of Leeds, United Kingdom of Great Britain and Northern Ireland

The reviews for this paper are both very positive, calling “very well researched” and its main arguments “strong, important, convincing and clearly conveyed”. The reviewers have suggested only minor changes to the manuscript. We are happy to accept the paper subject to these suggested minor revisions.

We believe this paper will make a strong contribution to this Special Issue. In a fascinating exploration of the epistemology of extinction, Tabak interrogates taxonomy and classification in both the scientific tradition and as a type of environmental storytelling in poetry. Tabak shows how listmaking may enrich poetry and facilitate conservation of biological species yet it simultaneously extends political and linguistic imperialism and “ignores and erases the biocultural worlds of which each species is a part.”

# Editor report: Decision: Taxonomies of exclusion: Storytelling, naming and classification in an age of extinction — R0/PR3

**DOI:** 10.1017/ext.2024.7.pr3

**Affiliations:** School of Natural Sciences, University of Tasmania, Australia

No accompanying comment.

# Editor report: Recommendation: Taxonomies of exclusion: Storytelling, naming and classification in an age of extinction — R1/PR5

**DOI:** 10.1017/ext.2024.7.pr5

**Affiliations:** School of Languages, Cultures and Societies, University of Leeds, United Kingdom of Great Britain and Northern Ireland

Thank you for making the (relatively minor) edits suggested by the reviewers. This paper will make a great contribution to the Special Issue!

Diane

# Editor report: Decision: Taxonomies of exclusion: Storytelling, naming and classification in an age of extinction — R1/PR6

**DOI:** 10.1017/ext.2024.7.pr6

**Affiliations:** School of Natural Sciences, University of Tasmania, Australia

No accompanying comment.
